# Miscellanea

**Published:** 1855-09

**Authors:** 


### MISCELLANEA.

*Deaths.*—M. Valleix, the author of the *Guide du Médicin Practicien*, *a Traité des Névralgies*, etc., died at Paris of malignant sore-throat on the 12th of July last.—At Richmond, Va., on the 15th of July, of cardiac disease, Dr. R. L. Bohannan, Professor of Obstetrics in the Medical College of Virginia, aged 64 years.—At Saratoga Springs, on the 20th of August, Dr. Moreton Stillé, in the 33d year of his age. Dr. S. was lecturer on the Theory and Practice of Medicine in the *Philadelphia Association for Medical Instruction.*—James Conquest Cross, M. D., died in Maysville, Kentucky, September (instant,) 3d.—Dr. James C. Bliss, one of the oldest physicians in New York, died in that city July 31st.—The yellow fever in Virginia has not abated. The number of deaths in Norfolk average 48 daily, and in Portsmouth 20. Dr. Stone, of New Orleans, pronounces it to be of the same type as that which afflicted New Orleans in 1853.—The fever is on the decline in New Orleans.

Dr. N. D. Benedict, late Superintendent of the New York State Lunatic Asylum, is about establishing in Florida a *Sanitarium* for affections of the throat and lungs. It is situated at Magnolia on the St. Johns River, between St. Augustine and Jacksonville, about twenty miles from the coast.

Dr. John W. Paxton has opened in Knoxville, Tennessee, an Infirmary under the name of Hotel for Invalids.

Dr. W. J. Holt, a young American surgeon, engaged in the Russian service in the Crimea, is winning golden opinions from the Northern Bear by the manner in which he is treating his wounded soldiers—and the golden opinions of the Emperor are almost equal to the ready cash.

Dr. Beale has been denied a new trial by the Supreme Court. There having been some informality or error in the sentence of the lower court, it has been ordered to give its decision according to law.

Madison, Ia., Aug. 21, 1855.

*The "Cut Direct."*—At a meeting of the Madison Medical and

Chirurgical Society, held on Monday evening last, after the transaction of the usual business of the Society, it was—

*Resolved*, Whereas, Drs. J. Jackson and T. W. Forshee have behaved themselves in such an unprofessional, ungentlemanly and contemptible manner towards the profession in general, that their names be erased from the record of the Society, and they be debarred from all prefessional intercourse with the members of the Society.

This resolution was passed unanimously.

Three-fourths of medical men are said to die before the age of fifty, and ten elevenths before sixty.

Allen & Co., publishers of the American Medical Advertiser, have a list of 40,000 Doctors.

Solon Borland, M. D., known in political circles as a democrat, a know nothing, and an ex-senator, and who was for a short season minister plenipotentiary to Central America, and destroyer (by proxy) of Greytown, has been appointed Professor of Physiology and Pathology in the Memphis Medical College.

Professor Agassiz will open a school for young ladies at his house in Cambridge on the 26th of September.

Prof. E. M. Moore has resigned the chair of Surgery in the Starling Medical College, and Prof. Hamilton, who before occupied the chair of Materia Medica and Therapeutics, has been appointed in his stead. H. L. Thrall, M. D., of Columbus, succeeds Dr. Hamilton.—S. Loving, M. D., has been appointed Demonstrator of Anatomy in place of Dr. McMillen, resigned.

Dr. Bulkley has retired from the New York Medical Times.

There are thirty-eight periodicals now existing in this country devoted to the interests of medicine.

Messrs. Blanchard & Lea have in preparation for early publication an "Introduction to Practical Pharmcy," by Prof. Parrish, of the Academy of Medicine; a new Dissector, entitled "The Practical Anatomist," by Prof. J. M. Allen, M. D., of the Pennsylvania Medical College; an elaborate treatise on "The Principles and Practice of Physical Exploration," by Professor Austin Flint, of Louisville; and a work on "Human Histology," by Professor Peaslee, of Dartmouth. Among their reprints we learn that they now have ready Rokitansky's "Pathological Anatomy," complete in two large volumes; Mackenzie on the Eye, with Dr.

Hewson's Annotations, in a splendid octavo of 1000 pages; Hoblyn's "Medical Dictionary," thoroughly revised and enlarged by Dr. Hays; and Tanner's "Manual of Clinical Medicine."

*The American Pharmaceutical Convention* will hold its annual session in the city of New York on the second Tuesday in September. Wm. B. Chapman, of Philadeldhia, is President for 1855.

*Massachusetts General Hospital.*—Dr. Jacob Bigelow has resigned his position as physician to the Massachusetts General Hospital, which he has held for nineteen years. His vacated place has been filled by the election of Dr. Augustus A. Gould.

*Boston Veterinary Institute.*—This Institution, incorporated by the Legislature, will commence its session in November next; the course of instruction to continue for four months. Its object is to afford ample instruction in the practice of veterinary medicine and surgery.

*Miami Medical College, Cincinnati.*—Prof. J. Davis has been appointed in place of Prof. John Avery, resigned.

In Boston five hundred subscribers have already been obtained for Agassiz's "Contributions to the Natural History of the United States."

*The Ohio State Medical Society*, at a recent meeting at Zanesville, O., rescinded, by an almost unanimous vote, the resolution adopted at its last annual meeting, viz: "That it is not derogatory to medical dignity, or inconsistent with medical honor, for medical men to take out a patent for medical or surgical instruments."

Dr. Peter Parker, for 20 years medical missionary in China, has been recently appointed United States Commissioner to the Chinese Court.

M. Jules Cloquet has been elected to fill the vacancy in the Section of Medicine and Surgery in the Academy of Sciences, occasioned by the decease of M. Lallemand. He was elected by one vote over M. Jobert.

The London Times, Punch, and sundry other papers in Great Britain, positively refuse to publish quack advertisements.

